# Management of bleeding in palliative care patients in the general internal medicine ward: a systematic review

**DOI:** 10.1016/j.amsu.2019.12.002

**Published:** 2019-12-18

**Authors:** R. Sood, M. Mancinetti, D. Betticher, B. Cantin, A. Ebneter

**Affiliations:** a Internal Medicine Department, Fribourg Hospital, Chemin des Pensionnats 2-6, 1752, Villars-sur-Glâne, Switzerland; b Medical Education Unit, University of Fribourg, Avenue de l'Europe 20, 1700, Fribourg, Switzerland; c Palliative Care Department, Fribourg Hospital, Avenue Jean-Paul II 12, 1752, Villars-sur-Glâne, Switzerland

## Abstract

**Background:**

Palliative care patients, those suffering from at least one chronic lifelong medical condition and hospice care patients, those with a life expectancy less than 6 months, are regularly hospitalised in general internal medicine wards. By means of a clinical case, this review aims to equip the internist with an approach to bleeding in this population. Firstly, practical advice on platelet transfusions will be provided. Secondly, the management of bleeding in site-specific situations will be addressed (from the ENT/pulmonary sphere, gastrointestinal - urogenital tract and cutaneous ulcers). Finally, an algorithm pertaining to the management of catastrophic bleeding is proposed.

**Methods:**

Electronic databases, including EMBASE, Pubmed, Google Scholar and the Cochrane Library were studied as primary resources, in association with local guidelines, to identify papers exploring platelet transfusions and alternative management of site-specific bleeding in palliative care patients.

**Results:**

Haemorrhagic complications are frequent in palliative care patients in the internal medicine ward. Current guidelines propose a therapeutic-only platelet transfusion policy. Nonetheless, prophylactic and/or therapeutic transfusion remains a physician-dependent decision. Site-specific therapeutic options are based on expert opinion and case reports. While invasive measures may be pertinent in certain situations, their application must be compatible with patient goals. Catastrophic bleeding requires caregivers' comforting presence; pharmacological management is secondary.

**Conclusion:**

Literature is lacking regarding management of bleeding in the palliative care population hospitalised in an acute medical setting. Recommendations are of limited quality, the majority based on case reports or expert opinion. Further studies, exploring for example the impact on patient quality of life, are desirable to improve the management of this frequently encountered complication.

## 1 Introduction

**Clinical case**: *A 49-year old male is admitted to the internal medicine ward complaining of a decline in general health. He was diagnosed with oesophageal cancer 6 months earlier and since has completed 6 cycles of chemoradiation. Personal history reveals increasing dysphagia with episodes of broncho-aspiration over the past 3 days, as well as significant weight loss since the initial diagnosis. Over the past week, he reports melaena (no haematemesis). The laboratory work-up reveals bicytopenia with haemoglobin at 90 g/l (norms 140–180 g/l), platelets 25 G/l (norms 150-400 × 10^9^/L), an inflammatory syndrome CRP 235 mg/dL (norms < 10 mg/dL), white blood cell count at 15.5 G/l (norms 4.0–10.0 G/l) and renal failure AKIN 1 (Creatinine 130 μmol/l, norms 50–110 μmol/L). This is the second hospitalisation since his initial diagnosis. The patient lives at home with his family and until recently, was independent forinstrumental and basic activities of daily living. He benefits from weekly visits from the mobile palliative unit, who recommended his current hospitalisation. You are the resident doctor on call for the weekend. The nurse calls you to inform you of recurrent episodes of melaena. Upon examination, the patient complains of orthostatic symptoms (dizziness and blurry vision upon sitting up), but no nausea or abdominal pain.*

General medicine wards play a pivotal role in the implementation of palliative care, as a high prevalence of admitted patients meet the criteria to qualify for palliative care [1]. Palliative care patients, defined as patients suffering from at least one chronic lifelong medical condition, and hospice care patients, defined as having a life expectancy less than 6 months, are regularly hospitalised in general internal medicine wards [2]. According to Pennell et al., one third of patients hospitalised in this setting are within their last year of life, while 10% will die during their current acute admission [3]. The management of bleeding in this population is a relevant question, which often arises in clinical practice. The following review aims to equip the internal medicine doctor with an approach to this patient population. Firstly, practical advice on platelet transfusions will be provided. Secondly, the management of bleeding in site-specific situations will be addressed, that is from the ENT/pulmonary sphere, the gastrointestinal - urogenital tract and malignant ulcers. Finally, an algorithm pertaining to the management of catastrophic bleeding is proposed.

All dose recommendations are adapted to be used in a non-specialized internal medicine ward setting; many of these are off-label. Most of the following recommendations are based on expert opinion. The application to concrete clinical settings depends on the availability of resources, cost-pertaining factors, and personal preference. Management must correspond to overall goals of care, which requires prior discussion in order to enable planned access to therapeutic and palliative measures. Invasive procedures will be mentioned and only further developed when considered useful.

## 2 Methods

Electronic databases, including EMBASE, Pubmed, Google Scholar and the Cochrane Library were used as primary resources, in association with local guidelines, in order to identify papers exploring platelet transfusions and alternative management of site-specific bleeding. References contained in selected articles were manually explored in search of additional papers of interest. The inclusion criteria regarding site-specific guidelines are detailed in respective tables. The aforementioned resources were explored using the following keywords: ‘palliative care’, ‘end-of-life’, ‘chronic progressive disease’, ‘incurable’, ‘platelet transfusion’, ‘haemorrhage’, ‘massive blood loss’, ‘major bleeding’ and ‘catastrophic bleeding’. English, French and German publications were considered for eligibility. Data selection was performed by RS and data extraction in duplicate (RS and AE); studies not conforming to the inclusion criteria were excluded. Risk of bias was explored at the outcome level. Research has been reported in line with the PRISMA [4] and the AMSTAR 2 criteria [5] (refer to supplementary material).

## 3 Platelet transfusions in palliative care patients

Transfusion practice has been subject to many paradigm shifts, especially the platelet transfusion threshold. Evidence necessary to establish transfusion guidelines is lacking [6], even though thrombocytopenia is often encountered in this population [7]. A national audit conducted amongst palliative care patients in the United Kingdom estimated that 28% of platelets are used outside of guidelines [8].

**Fig. 1:**
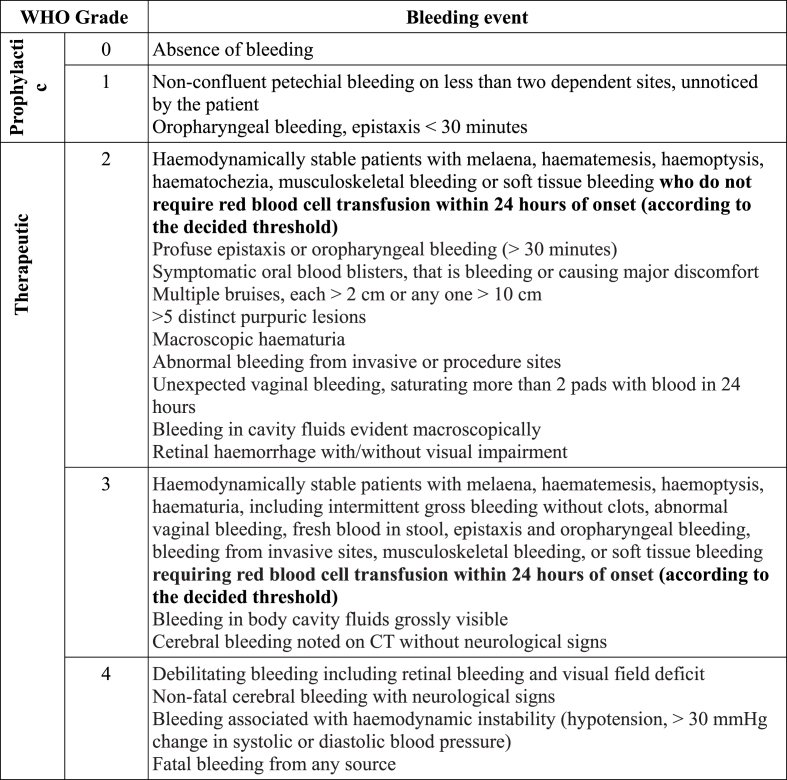
**Modified WHO Bleeding Score Classification, adapted from** Refs. [9,10].

Bleeding is defined as per the WHO bleeding score classification (Fig. 1). This universal tool allows for the distinction of platelet transfusion as **prophylactic**, defined as WHO grade 0–1 or **therapeutic**, defined as WHO grade 2 and above.

Various factors correlate with an increased risk of bleeding independently of platelet count (Table 1). Platelet count provides no qualitative information about the risk of haemorrhage. Platelet transfusion should always be accompanied by an optimisation of the coagulation system and withdrawal of anticoagulant and anti-platelet drugs, as well as drugs with anticoagulant side-effects. Basic measures, including those employed to reduce the visual impact of bleeding and alleviate the suffering of the family, cessation of exacerbating factors, including treatment of infection and positioning of the patient, are recommended in all situations and hence will not be further discussed in this paper. Comorbidities considered significant and applicable to the general population, are outlined in Table 1.

**Table 1:** Factors associated with an increased bleeding risk, adapted from Ref. [11].

**Factors associated with an increased risk of bleeding**
Recent blood and bone marrow haematopoietic stem cell transplantation
Recent history of severe haemorrhage (≤5 days)
Infection
Treatment related causes, drugs
Malnutrition
Underlying disease (graft versus host disease, venoocclusive disease, cancer, cirrhosis)

### 3.1 Prophylactic platelet transfusions (WHO bleeding event grade 0–1)

An organizational survey conducted in 2012 determined that platelet transfusions are prophylactic interventions in 69% of cases [8]. Furthermore, in a retrospective study from 2013 approximately 50% of haematological inpatients received a platelet transfusion for bleeding prophylaxis in their final week of life [12]. This is significant when considering that 40% of hospice organisations in the United States prohibit transfusion support as per a national survey conducted in 2009 [13], most probably due to financial considerations. A 2017 American study estimated the total direct cost of a platelet transfusion in chronic liver disease patients with thrombocytopenia between $5258 to $13,117 (2017 US dollars) [14]. While the financial impact of these statistics is staggering, it further presents a clear obstacle in terms of the allocation of resources.

Although prophylactic platelet transfusions are often performed, few studies exploring this strategy exist. Estcourt et al. explored the platelet transfusion threshold in prophylactic transfusions by means of three randomised controlled trials. This study provided low quality evidence to support a transfusion trigger level of 10 × 10^9^/L in ≤ grade 2 bleeding [15].

### 3.2 Therapeutic platelet transfusions (WHO bleeding event grade ≥ 2)

A Cochrane systematic review updated in 2015 did not show any difference in all-cause 30-day mortality or adverse events between a therapeutic-only versus prophylactic platelet transfusion policy [16]. The OPTIMAL pilot trial, which aimed to investigate the occurrence of bleeding events in patients receiving therapeutic or prophylactic transfusions in this population, was discontinued due to insufficient recruitment, which highlights the challenges encountered in such studies [17]. Studies are lacking comparing these transfusion strategies, especially those exploring the impact of transfusion policy on patient quality of life.

Due to the existence of confounding comorbidities and the paucity of evidence, indications regarding whether to transfuse platelets in prophylactic or therapeutic situations are subject to debate. Current guidelines nonetheless propose a therapeutic-only transfusion policy in palliative care patients [6,9].

In conclusion, the approach to platelet transfusion, that is prophylactic versus therapeutic, is a decision in a clinical setting to be made with the patient. The same applies to the platelet transfusion threshold, again due to the lack of randomized controlled trials. On the basis of existing evidence, we propose the transfusion strategy, summarised in Table 2.

**Table 2:** Proposed platelet transfusion strategy for palliative care patients hospitalised in an internal medicine ward, based on [9]and expert opinion.

ECOG performance status		Active treatment	Platelet transfusion threshold (G/l), *norms 150–*400 G/L	
			Prophylactic (WHO bleeding scale 0–1)	Therapeutic (WHO bleeding scale ≥ 2)
	**0**–**2 (good)**	Yes	10 (20^a^)	30 (Target range 30–50)
		No	10 (20^a^)	
	**3**–**4 (poor)**	Yes	10	
		No	Individualised/Exceptional	
		End-of-life	Not recommended	Individualised/Exceptional

a 20 in certain comorbidities (Table 1) with increased bleeding risk or systemic inflammation.

**Follow-up of clinical case**: *This clinical case presents a situation commonly encountered in the internal medicine ward. Personal history reveals many factors associated with an increased risk of bleeding, a possible infectious context and treatment-related causes (chemoradiation). The platelet transfusion threshold will be determined by the performance status of the patient, which is presumed to be “good” (living at home, independent for daily activities). He presents grade 2 bleeding and so the proposed platelet transfusion threshold would be of* 30 G/l*. In the presence of bleeding classified as WHO bleeding scale 0–1 a prophylactic platelet transfusion would be proposed with a threshold of 20 G/l*.

## 4 Recommendations for management of site-specific bleeding

Clinically significant bleeding occurs in 6–10% of palliative care patients [18]. The following section will aim to explore treatment options in site-specific bleeding complementary to platelet transfusions. As previously mentioned, complementary measures including the withdrawal of anticoagulant and anti-platelet drugs, as well as drugs with anticoagulant side-effects must be adopted.

### 4.1 Head and neck pathologies

Anatomical reasons explain the prevalence of bleeding in head and neck cancers; it has been described that 74% of this population experienced bleeding during their final month of life [19]. The accessibility of the actively bleeding lesion is key when determining management. Patients suffering from accessible actively bleeding lesions are candidates for local therapy.

In the nasopharynx, silver nitrate sticks can be used [20,21]. Topical gauze soaked in tranexamic acid inserted into the nostril for 10 min is a viable therapeutic option for anterior epistaxis [22,23], whereas posterior epistaxis responds to sympathomimetic vasoconstrictors [24,25].

In the oropharynx, lesions are susceptible to mouthwashes (tranexamic acid or sucralfate) [[26], [27], [28], [29]], while nebulisations can be used for less accessible lesions (adrenaline) [30]. Therapeutic options for inaccessible lesions are limited to systemic tranexamic acid [31].

Endovascular techniques can be considered for bleeding related to vascular erosion: embolisation has been shown to be highly efficient yet not devoid of complications [32,33]. Radiotherapy is another viable option [32], many patients have however already received maximal doses of radiotherapy excluding further irradiation [33]. Surgical management may be considered if conservative measures fail in a selected population, as explored in existing descriptive studies. No studies regarding the use of palliative chemotherapy in bleeding management were found.

**Summary** (See also Appendix A and B): Anterior nasopharyngeal bleeding can be treated with silver nitrate sticks or local tranexamic acid. Posterior nasopharyngeal bleeding can be treated with sympathomimetic vasoconstrictors. Oropharyngeal bleeding accessible lesions are candidates for tranexamic acid or sucralfate mouthwash, while less accessible lesions are candidates for adrenaline nebulisations. Inaccessible ENT lesions require systemic tranexamic acid. If compatible with patient attitude, embolisation, radiotherapy and surgical management can be considered in selected populations.

### 4.2 Pulmonary bleeding

The frequency of haemoptysis in the course of pulmonary cancer, is estimated at 20%, while massive terminal haemoptysis at 3% [34].

Invasive strategies are indicated in the management of potentially reversible bleeding; this must be considered following overall evaluation of the patient's attitude. Bronchoscopy (flexible or rigid) performed urgently allows for blood clot removal and mechanical tamponade [35]. Such an intervention can also allow for cold-saline lavage or laser phototherapy [36]. Such interventions provide temporary respite. Meanwhile, the use of palliative thoracic irradiation for various symptoms, including haemoptysis, is recognised [37].

As massive haemoptysis originates in 90% of cases from the high-pressure bronchial circulation [36], bronchial artery embolisation is the most effective non-surgical alternative, with a recurrence rate of approximately 15% and immediate success rate high as 98% [38].

The place of medication in the management of haemoptysis remains abstract, with few studies addressing the question. A Cochrane study exploring the role of antifibrinolytic therapy did not find sufficient evidence pertaining to it's use in the management of pulmonary bleeding [39]. The most commonly used antifibrinolytic agent is tranexamic acid [21]. Consensus is lacking regarding the recommended route of administration. Although intravenous administration remains the most studied [[40], [41], [42]], a recently published randomised controlled trial confirmed the topical use of tranexamic acid as a safe and promising alternative [[43], [44], [45]].

Initially developed for coagulopathies, the off-label use of recombinant FVIIa in non-haemophiliac patients is increasing. This review will not include this treatment in the following recommendations due to the expense and requirement for intensive care surveillance.

**Summary** (See also Appendix A and B): Topical tranexamic acid is a viable therapeutic option, systemic administration can also be considered. Invasive measures, including bronchoscopy or embolisation, must be considered according to patient attitude. Palliative radiotherapy for low grade chronic haemoptysis is an option.

### 4.3 Gastrointestinal bleeding

Mercadante et al. showed that in homebound palliative care patients the incidence of gastrointestinal bleeding is 2.25%. Bleeding proved fatal in 56% of patients within 48 hours [46]. For bleeding originating proximal to the ligament of Treitz, initial management requires proton pump inhibitors and urgent gastroscopy (if the situation is not considered terminal). The oral administration of antifibrinolytic agents was explored in a 2014 Cochrane review [47], that concluded that the use of tranexamic acid in upper gastrointestinal bleeding has a beneficial effect on mortality. However, this effect was not considered statistically significant. The HALT-IT trial (currently underway), exploring the use of tranexamic acid in upper or lower gastrointestinal bleeding, aims provide further information [48].

Certain vasoactive agents counter upper gastrointestinal bleeding through portal pressure reduction, including vasopressin and octreotide. Retrospective studies, which date from 1990, suggest that vasopressin is an effective treatment in neoplastic-related gastrointestinal bleeding [49]. However, due to its adverse effect profile, it is less used. Somatostatin and it's analogue, octreotide, are on the other hand associated with fewer side effects [50].

While the use of sucralfate has been shown to treat gastric erosions [51] and prevent stress ulcers in intensive care patients [52], no studies were found exploring the use of sucralfate suspension in bleeding management in palliative care patients.

Evidence regarding lower gastrointestinal bleeding pharmacological management in palliative care patients is anecdotal, based on case reports. Rectal bleeding can benefit from local treatments, including packing. The use of rectal tranexamic acid was initially reported in a case report in 1991 [53]. Sucralfate enema in haemorrhagic radiation proctitis was initially explored by Kochhar et al. [54]. More recent studies propose the use of sucralfate paste instead of enema administration, due to the difficulty in retaining the enema suspension in the case of active proctitis.

Colonoscopic access to the site of lower gastrointestinal tract bleeding can allow for laser treatment, cryotherapy, argon plasma coagulation or packing, yet may be challenging [55]. Endoscopic management in patients with adequate performance status is nonetheless associated with high levels of morbidity [56]. Furthermore, recurrent bleeding following endoscopic haemostasis is frequent.

In the case of failure to achieve haemostasis through endoscopic intervention in upper or lower gastrointestinal bleeding, palliative haemostatic radiotherapy should be considered. Such treatment is considered to provide durable palliation of bleeding in gastric cancer [57,58]. Angiography, embolisation and surgery should also be considered while taking into consideration the situation of the patient [59]. Evidence remains however anecdotal.

**Summary** (See also Appendix A and B): Systemic tranexamic acid is a therapeutic option for gastrointestinal bleeding. Somatostatin (or octreotide) is to be considered in upper gastrointestinal bleeding. Lower gastrointestinal bleeding can also respond to rectal administration of sucralfate paste or tranexamic acid. Endoscopic management should be discussed according to patient attitude. Alternative invasive management includes embolisation. In case of failure, palliative haemostatic radiotherapy should be considered.

**Follow up of clinical case:** *Our patient is a candidate for tranexamic acid administration. Invasive management is to be considered in light of the good performance status of the patient (endoscopic intervention with haemostasis). Following the exclusion of signs in favour of active thromboembolic disease, tranexamic acid 10 mg/kg b.i.d. administration can be proposed, which corresponds to 450 mg twice daily following adaptation to renal function (as per recommendations proposed by* Ref. [60]. *The patient initially presented no side effects (notably, no nausea, vomiting or diarrhoea). The patient developed colour vision disturbance, which required discontinuation of the drug. Following cessation of the drug, there was no recurrence of bleeding and normal vision was spontaneously restored, discharge was possible one week later with reactivation of ambulatory services.*

### 4.4 Urogenital bleeding

Management of bleeding originating from the urogenital tract depends on the origin. The following subsection will thus respect the anatomical regions of the bladder, prostate and vagina, in order to address the management of bleeding in palliative care patients.

### 4.5 Vesical bleeding

Intractable haematuria is considered a common complication in patients with inoperable bladder carcinoma [61]. The initial approach involves large three-way Foley urethral catheter placement, allowing for decompression and saline irrigation [62,63]. Due to possible ischaemic side effects and secondary bleeding from the urothelium, this intervention should be limited in duration [62]. It is significant to note that intractable haematuria linked to inoperable bladder cancer often fails to respond such interventions [61].

Various agents can be injected intra-vesically. While studies have shown formalin to be the most effective intra-vesical haemostatic agent, it's administration requires anaesthesia (general or spinal) and a cystogram to exclude vesico-ureteral reflux or bladder perforation. Furthermore, the adverse effect profile limits it's application in practice [64]. This review does not consider formalin as a viable therapeutic option in palliative care patients. Other treatments, such as intravesical instillations of carboprost tromethamine and conjugated oestrogens, have been explored in case reports but do not have widespread utility in light of the lack of evidence [[65], [66], [67]].

Oral administration of antifibrinolytic agents, tranexamic acid or aminocaproic acid, involves a risk of clot retention due to inhibition of fibrinolysis of pre-formed fibrin deposits. Clot removal requires general anaesthesia and thus, such treatment is only to be considered as a last resort. Intravesical administration of antifibrinolytic agents can be considered in the case of refractory haematuria [68].

Various case-series and case reports explore invasive palliation of intractable haematuria in bladder cancer. Current guidelines propose cystoscopy with laser coagulation and resection of the bleeding site if compatible with the performance status of the patient [68]. Endovascular techniques have been shown to have a 90% bleeding response rate [64]. Such techniques however are not devoid of complications (including post-embolisation syndrome, transient voiding dysfunction and transient gluteal claudication) [61]. Other prospective studies are lacking.

Radiotherapy has been shown to be a safe and effective measure in palliative care patients. A prospective study published in 2016 including 44 patients showed a mean haematuria-free follow-up of 13 months [69].

### 4.6 Prostate bleeding

In prostate cancer, haematuria is the result of neoplastic invasion of the urethra and has been shown to be a leading cause of hospitalisation [70]. Hence, the insertion of a Foley catheter with mild traction may halt bleeding, as explored in the previous subsection [71]. Acute bleeding can be managed by oral tranexamic acid [31].

Endovascular management (transcatheter arterial embolisation), as studied in case reports, is considered a minimally invasive procedure in symptom palliation following the failure of conservative management of intractable haematuria originating from the prostate [72]. In a systematic review regarding palliative pelvic radiotherapy for symptom relief, pooled results showed a 73% reduction of haemorrhage [73]. Radiotherapy is an acceptable treatment, although the risk of radiation-induced cystitis should not be underestimated [74].

### 4.7 Vaginal bleeding

Cervical cancer is complicated by fatal vaginal bleeding in 6% of women [75]. An immediate simple measure is vaginal packing with gauze rolls in a lithotomy position, which may stop bleeding through tamponade.

Anecdotal case studies exist regarding the topical application of Mohs’ paste (zinc chloride) and Monsel solution (ferric subsulphate) [76]. Although studies mention the use of oral tranexamic acid, no dedicated studies to support this recommendation were found [77,78]. The use of antifibrinolytic agents in palliative care patients suffering from vaginal bleeding thus remains unclear, but we consider it a possible treatment option.

Invasive procedures such as uterine artery embolisation using radiological techniques have been used [77]. Transvaginal radiotherapy may also be considered with an acceptable adverse effect profile as shown in a prospective trial published in 2005 [79].

**Summary** (See also Appendix A and B): Intravesical administration of antifibrinolytic agents is the mainstay of treatment of vesical bleeding. According to the ECOG performance status, cystoscopy or radiotherapy may be considered. Oral tranexamic acid should be administered for prostate bleeding. Invasive modalities include endovascular management or radiotherapy. Vaginal bleeding responds to local tamponade. Although antifibrinolytic agents have not been studied, their use may be considered. Invasive techniques for the latter include embolisation or local radiotherapy.

### 4.8 Cutaneous ulcer bleeding

Wound care can be considered to be both a curative and palliative intervention. The physiology of healing is altered in terminal disease, bleeding may result from blood vessel erosion or be trauma-induced during wound dressing. The burden on the quality of life may be important [80,81]. Therapeutic options, other than compression, are mainly topical and knowledge regarding haemostatic properties of the following proposed substances are based on expert opinion.

The use of alginate dressings in malignant ulcers is off-label in spite of known haemostatic properties. Alginate dressing removal tends to provoke mechanical damage and thus subsequent haemorrhage [82]. Other local treatments include tranexamic acid [83,84] or (short-term) adrenaline-soaked gauze. The systemic effect of the topical application of such substances, especially if in contact with mucosae, remains uncertain. Anecdotal evidence exists regarding sucralfate application and Mohs’ paste [85,86]. Oral antifibrinolytics can be considered to prevent further bleeding [31].

Invasive techniques include transcatheter or transcutaneous (that is direct) embolisation [87] and radiotherapy [82]. Debulking surgery can be considered when radiation options have been exhausted, but often the overall patient profile compromises this option in palliative care patients.

**Summary** (See also Appendix A, Appendix B): Alginate dressings, local tranexamic acid or adrenaline soaked gauze are validated topical treatments. Oral antifibrinolytics can be considered to prevent further bleeding. Embolisation or radiotherapy may be considered according to overall patient attitude.

## 5 Catastrophic bleeding

Catastrophic bleeding is defined as grade 5 bleeding, as per the WHO bleeding score classification, which results in imminent death (within minutes). It is a retrospective diagnosis, as patients may experience sentinel bleeds that do not result in terminal haemorrhagic events. The incidence has been reported to vary, with the highest incidence reported in patients with ENT cancers and complications such as carotid blow-out described to be as high as 4% [88,89].

The identification of patients at risk is primordial in anticipating potential outcomes and initiating supportive measures [90]. Various factors, such as those underlined in Table 1, are to be considered. Initial approach to the event requires the identification of the source of bleeding and application of pressure, if appropriate. During a catastrophic bleed, reassurance and comfort are of utmost importance. Positioning can be modified, for example in the case of massive haemoptysis, placement of the patient in a lateral decubitus position towards the site of bleeding aims to avoid aspiration to the non-affected side and avoid suffocation. In many situations, pharmacological management may not be effective due to the rapidity of onset and degradation.

Current practice advocates the use of sedatives as the pharmacological management of catastrophic bleeding. Such medication, most commonly *Midazolam* 5 mg intravenous or intranasal, aims to reduce awareness and distress [21,91]. Certain guidelines propose the use of opioids. This review recommends the use of opioids only in the case of overt pain or dyspnoea. Furthermore, Harris et al. conducted a qualitative study regarding caregiver perception of crisis medication; focus on pharmacological management was shown to be at the detriment of supportive measures [88]. Pharmacological management should therefore not detract from non-pharmacological approaches.

It is essential that a crisis plan of action be established and goals of care clarified. The question whether life support is desired must be addressed prior to an eventual event. In patients who are identified as being at risk, a bedside crisis pack should be available containing prepared sedatives, while carefully considering the psychological impact (reminder of risk) and the pharmacological stability [92].

## 6 Discussion

Platelet transfusion strategy should be based on an individualised decision, while considering patient performance status and the WHO bleeding event grade. The heterogeneity of the patient population hospitalised in the internal medicine ward does not allow for the application of a therapeutic-only platelet transfusion policy as proposed by Refs. [6,9]. Therefore, a clinical based approach is proposed, which requires taking the overall performance status of the patient into consideration (Table 2).

Site-specific bleeding is a frequent complication encountered in palliative care patients. While invasive measures may be pertinent in certain situations, their adoption requires access to certain resources and must be compatible with the patient's goals of care. Primary management must include non-pharmacological measures, as well as local pharmacology when appropriate. Systemic intervention is usually secondary.

Patients at risk of catastrophic bleeding must be identified and goals of care clarified. Attempts must be made to anticipate potential outcomes, such as catastrophic situations. The mainstay of management requires the healthcare professional's presence. Pharmacological management is secondary; when crisis medication is appropriate and does not detract from the latter, *Midazolam* is the medication of choice to achieve sedation.

## 7 Limitations

Literature is lacking regarding the management of bleeding in an acute medical setting, as outlined in this review. The quality of the described recommendations is limited, as the majority of research is based on case reports or expert opinion. The implementation of such studies in the palliative care population are difficult and risk of basis is difficult to evaluate. Many obstacles exist, while the subject of such studies is highly sensitive, funding and institutional capacity have also been identified as prominent obstacles to palliative care studies [93].

## 8 Conclusion

The growing prevalence of the palliative care patient population with specific needs means that further studies are necessary in order to ameliorate management in at-risk patients. Such studies may consider exploring for example the impact of bleeding management on patient quality of life.

## Ethical approval

Not relevant.

## Sources of funding

None.

## Author contribution

All authors were involved in the study conception and design. R. Sood wrote the manuscript, which was then revised and edited by all authors.

## Trial registry number

https://www.researchregistry.com/

Research Registry UIN: reviewregistry748.

## Guarantor

R. Sood.

## Consent

Not relevant.

## Declaration of competing interest

None.

## Figures and Tables

**Table:** 

Lesions situated in the nasopharynx					
Therapy	Posology	Administration	Adverse effects	Contra-indications	Ref.
Silver nitrate	75% AgNO_3_ 25% KNO_3_	Topical (sticks)	Pain Skin irritation Septal perforation Methemoglobinemia (rare)	?	[20,21]
Xylometazoline	1:1000	Nasal spray	Rebound vasodilation (if prolonged use)	Closed-angle glaucoma	[24,25]
Tranexamic acid	500 mg/5 ml	Topical (gauze), for 10 min	?	?	[22,23]
**Lesions situated in the oropharynx**					
**Therapy**	**Posology**	**Administration**	**Adverse effects**	**Contra-indications**	**Ref.**
Tranexamic acid	500 mg/5 ml, 10 mL q.i.d.	Mouthwash suspension (swallow after use)	Nausea, vomiting and diarrhoea (25%) Colour vision disturbance	Dose reduction if GFR < 50 ml/mn Active thromboembolic disease	[[26], [27], [28], [29]]
Adrenaline	2.5–5 ml 1% in 5 ml 0.9% saline q.i.d.	Nebulisation	None	None	[30]
**Inaccessible lesions (ENT)**					
**Therapy**	**Posology**	**Administration**	**Adverse effects**	**Contra-indications**	**Ref.**
Tranexamic acid	1–1.5 g t.i.d.	Oral	Nausea, vomiting and diarrhoea (25%) Colour vision disturbance	Dose reduction if GFR < 50 ml/mn Active thromboembolic disease	[31]
	10–15 mg/kg t.i.d.	Intravenous			
**Pulmonary pathologies**					
**Therapy**	**Posology**	**Administration**	**Adverse effects**	**Contra-indications**	**Ref.**
Tranexamic acid	500 mg/5 ml t.i.d. - q.i.d.	Nebulisation	None	?	[[43], [44], [45],^60^]
	1–1.5 g t.i.d.	Oral	Nausea, vomiting and diarrhoea (25%) Colour vision disturbance	Dose reduction if GFR < 50 ml/mn Active thromboembolic disease	[39]
	10 (−15) mg/kg t.i.d. - q.i.d.	Intravenous			[[40], [41], [42]]
**Upper gastrointestinal bleeding**					
**Therapy**	**Posology**	**Administration**	**Adverse effects**	**Contra-indications**	**Ref.**
Tranexamic acid	1–1.5 g t.i.d.	Oral	Nausea, vomiting and diarrhoea (25%) Colour vision disturbance	Dose reduction if GFR < 50 ml/mn Active thromboembolic disease	[47]
	10–15 mg/kg t.i.d.	Intravenous			
Octreotide	100–200 mg t.i.d.	Subcutaneous	?	None	[50]
	10 mcg push then 25 mcg/h	Intravenous	If > 100 mcg/h: nausea, abdominal discomfort, diarrhoea		
**Rectal bleeding**					
**Therapy**	**Posology**	**Administration**	**Adverse effects**	**Contra-indications**	**Ref.**
Tranexamic acid	500 mg, 10 ml water b.i.d.	Rectal (enema)	?	?	[53]
	1 g t.i.d.	Oral	Nausea, vomiting and diarrhoea (25%) Colour vision disturbance	Dose reduction if GFR < 50 ml/mn Active thromboembolic disease	[31]
**Vesical bleeding**					
**Therapy**	**Posology**	**Administration**	**Adverse effects**	**Contra-indications**	**Ref.**
Tranexamic acid	1 g t.i.d.	Oral	Nausea, vomiting and diarrhoea (25%) Colour vision disturbance	Dose reduction if GFR < 50 ml/mn Active thromboembolic disease	[60]
Tranexamic acid	100 ml of 5% b.i.d.	Intravesical	Intravesical clot retention	?	[60,68]
**Prostate bleeding**					
**Therapy**	**Posology**	**Administration**	**Adverse effects**	**Contra-indications**	**Ref.**
Tranexamic acid	1–1.5 g t.i.d.	Oral	Nausea, vomiting and diarrhoea (25%) Colour vision disturbance	Dose reduction if GFR < 50 ml/mn Active thromboembolic disease	[31]
**Vaginal bleeding**					
**Therapy**	**Posology**	**Administration**	**Adverse effects**	**Contra-indications**	**Ref.**
Tranexamic acid	500 mg/5 ml	Topical (gauze), apply pressure for 10 min	?	?	None
	1–1.5 g t.i.d.	Oral	Nausea, vomiting and diarrhoea (25%) Colour vision disturbance	Dose reduction if GFR < 50 ml/mn Active thromboembolic disease	None
**Malignant ulcers**					
**Therapy**	**Posology**	**Administration**	**Adverse effects**	**Contra-indications**	**Ref.**
Alginate	–	Topical dressings	Mechanical damage upon dressing removal	?	[60,82]
Adrenaline	1:1000	Topical (gauze) for 5–10 min	Rebound bleeding Ischaemic necrosis	None	[60]
Tranexamic acid	500 mg in 5 ml	Topical (gauze)	?	?	[83,84]
	1 g b.i.d. for 1 week following cessation of bleeding	Oral	Nausea, vomiting and diarrhoea (25%) Colour vision disturbance	Dose reduction if GFR < 50 ml/mn Active thromboembolic disease	[83,84]

Remarks: “?” corresponds to information currently unavailable in existing literature.

**Table:** 

Lesions situated in the oropharynx					
Therapy	Posology	Administration	Adverse effects	Contra-indications	Ref.
Sucralfate	2 g in 10 ml suspension b.i.d.	Mouthwash suspension	Unknown	?	[54]
**Pulmonary pathologies**					
**Therapy**	**Posology**	**Administration**	**Adverse effects**	**Contra-indications**	**Ref.**
Vasopressin	5UI in 2 ml saline	Nebulisation	Abdominal cramping Diarrhoea Hypernatremia Myocardial infarction Ectopic beats Mild hypertension	Hypersensitivity Cardiovascular disease Uraemic chronic renal disease Migraines Uncontrolled epilepsy Severe asthma	[40]
**Vesical bleeding**					
**Therapy**	**Posology**	**Administration**	**Adverse effects**	**Contra-indications**	**Ref.**
Aluminium salts	1% 50 g alum, 5L sterile water, 250 ml/h	Bladder irrigation	Vesical tenesmus Systemic toxicity (rare) Recurrence (frequent)	?	[60]
Conjugated oestrogens	12.5 mg–50 mg b.i.d. for 2–12 days 2.5 mg–20 mg b.i.d.	Intravenous followed by: Oral	Liver dysfunction, hypercoagulability, hypertension, malignant transformation	None.	[67]
**Vaginal bleeding**					
**Therapy**	**Posology**	**Administration**	**Adverse effects**	**Contra-indications**	**Ref.**
Sucralfate	2g in 10 ml	Topical	Unknown	?	None
Mohs' paste	50g zinc chloride, 25 ml water, 19g zinc starch, 15 ml glycerol	Topical	Pain	?	[94]
Monsel's paste	15g ferric subsulphate, ferrous sulphate power (a few grains), 10 ml water, 12g glycerol starch	Topical	Caustic effect on tissues	?	
**Upper gastrointestinal bleeding**					
**Therapy**	**Posology**	**Administration**	**Adverse effects**	**Contra-indications**	**Ref.**
Vasopressin	0.04 UI/min	Intravenous	Colic, diarrhoea, nausea (common) Worsening liver function in cirrhosis Ischaemia (portal thrombosis)	Use with caution in case of coronary artery disease, heart failure, vascular disease, chronic nephritis with nitrogen retention, water intoxication, migraine, seizure disorder, asthma	[21,49]
**Rectal bleeding**					
**Therapy**	**Posology**	**Administration**	**Adverse effects**	**Contra-indications**	**Ref.**
Sucralfate	Many 2g tablets, gel* b.i.d.	Rectal	Constipation	?	[54]
**Malignant ulcers**					
**Therapy**	**Posology**	**Administration**	**Adverse effects**	**Contra-indications**	**Ref.**
Sucralfate	1 g paste, 5 ml water, gel b.i.d.	Topical (gauze)	Unknown	?	[28,86]
Mohs' paste	50g zinc chloride, 25 ml water, 19g zinc starch, 15 ml glycerol	Topical	Pain	?	[85]

Remarks: “?” corresponds to information currently unavailable.

Provenance peer review.

Not commissioned, externally peer reviewed.

## Abbreviations

q.i.d.: once a day
b.i.d.: twice a day
t.i.d.: three times a day
q.i.d.: four times a day; SIRS systemic inflammatory response syndrome; ENT ear-nose-throat; ECOG Eastern Cooperative Oncology Group
GFR: Glomerular filtration rate
